# Angiogenic microspheres promote neural regeneration and motor function recovery after spinal cord injury in rats

**DOI:** 10.1038/srep33428

**Published:** 2016-09-19

**Authors:** Shukui Yu, Shenglian Yao, Yujun Wen, Ying Wang, Hao Wang, Qunyuan Xu

**Affiliations:** 1 Department of Neurobiology, Beijing Institute for Brain Disorders, Beijing Center of Neural Regeneration and Repair, Beijing Key Laboratory of Major Brain Disorders, Capital Medical University, 10 Xitoutiao, You An Men, Beijing 100069, China; 2 School of Materials Science and Engineering, University of Science and Technology Beijing, 30 Xueyuan Road, Beijing 100083, China; 3 School of Materials Science and Engineering, Tsinghua University, Hai Dian, Beijing 100084, China; 4 Ningxia Key Laboratory of Cerebrocranial Diseases, Department of Anatomy, Histology and Embryology, School of Basic Medical Sciences, Ningxia Medical University, 1160 Shengli Street, Yinchuan 750004, China; 5 Department of Anatomy, School of Basic Medical Sciences, Capital Medical University, 10 Xitoutiao, You An Men, Beijing 100069, China

## Abstract

This study examined sustained co-delivery of vascular endothelial growth factor (VEGF), angiopoietin-1 and basic fibroblast growth factor (bFGF) encapsulated in angiogenic microspheres. These spheres were delivered to sites of spinal cord contusion injury in rats, and their ability to induce vessel formation, neural regeneration and improve hindlimb motor function was assessed. At 2–8 weeks after spinal cord injury, ELISA-determined levels of VEGF, angiopoietin-1, and bFGF were significantly higher in spinal cord tissues in rats that received angiogenic microspheres than in those that received empty microspheres. Sites of injury in animals that received angiogenic microspheres also contained greater numbers of isolectin B4-binding vessels and cells positive for nestin or β III-tubulin (*P* < 0.01), significantly more NF-positive and serotonergic fibers, and more MBP-positive mature oligodendrocytes. Animals receiving angiogenic microspheres also suffered significantly less loss of white matter volume. At 10 weeks after injury, open field tests showed that animals that received angiogenic microspheres scored significantly higher on the Basso-Beattie-Bresnahan scale than control animals (*P* < 0.01). Our results suggest that biodegradable, biocompatible PLGA microspheres can release angiogenic factors in a sustained fashion into sites of spinal cord injury and markedly stimulate angiogenesis and neurogenesis, accelerating recovery of neurologic function.

Spinal cord injury can lead to complete and permanent loss of neurologic function. A cascade of pathophysiological events, including loss of blood supply, inflammation, and demyelination, contribute to a poor microenvironment at the injury site, jeopardizing neural regeneration and restoration^1^,^2^. After acute injury in the central nervous system (CNS), immature neuronal migration and axonal sprouting occur along neovessels in perilesional tissue^3^,^4^, suggesting close association of neurogenesis with angiogenesis in areas undergoing active neural tissue remodelling.

Vascular endothelial growth factor (VEGF), angiopoietin-1 (Ang-1) and basic fibroblast growth factors (bFGF) are major angiogenic factors in tissue growth and repair^5^. They promote neural regeneration and recovery of neurologic function following spinal cord injury. VEGF regulates endothelial cell proliferation, differentiation and growth in early angiogenesis, while Ang-1 contributes to migration, stabilization and maturation of blood vessels in late angiogenesis^6^. Wildenfalk *et al*. found that acute VEGF administration increased the amount of spared tissue and blood vessel density at sites of spinal cord contusion injury in rats^7^. des Rieux *et al*. demonstrated that local delivery of VEGF in an injectable, alginate/fibrinogen-based hydrogel stimulated angiogenesis and neurite growth in traumatized spinal cord in rats^8^. Herrera *et al*. showed that sustained delivery of both VEGF and Ang-1 to the lesion epicenter using adeno-associated viruses improved locomotor recovery of rats with traumatic spinal cord injury^9^.

The factor bFGF also contributes to early angiogenic responses^10^ and promotes neurologic function recovery following spinal cord injury. Rabchevsky *et al*. showed that intrathecal infusion of bFGF following severe contusion injury at T10 in rats significantly restored gross hindlimb motor function^11^. Hydrogel of hydroxyl ethyl methacrylate [2-(methacryloyloxy)ethyl] trimethylammonium chloride (HEMA-MOETACL) containing bFGF promoted both nerve tissue regeneration and functional recovery in a rat model of spinal cord injury^12^. These findings suggest that increasing the local concentration of angiogenic factors at the epicenter of spinal cord injury may promote angiogenesis, neural regeneration and neurologic functional recovery^9^,^13^.

Encapsulating angiogenic factors in polymer-based microspheres can allow their sustained release and maintenance at injury sites because the polymers are degraded slowly^14^. Microspheres based on poly(lactic-co-glycolic acid) (PLGA) are widely used due to the polymer’s biocompatibility and biodegradability, which allow controlled, stable release of drug^15^,^16^. In previous work with an animal model of stroke, our laboratory demonstrated that PLGA microspheres showed sustained release of growth factors, promoted neural stem cell (NSC) activity in the long term and improved neural recovery^17^,^18^. We also showed that PLGA microspheres loaded with brain-derived neurotrophic factor and VEGF promoted neoangiogenesis and neural regeneration in a rat dorsal hemisection model^19^.

Given evidence that VEGF, Ang-1 and bFGF promote angiogenesis and neurogenesis following spinal cord injury, we speculated that using PLGA microspheres to simultaneously release all three factors at sites of spinal cord injury may create a microenvironment more conducive for angiogenesis and neural regeneration, thereby promoting recovery of neurologic function. Here we investigated the effects of microsphere-mediated co-delivery of VEGF, Ang-1 and bFGF on vessel formation, neural regeneration and hindlimb motor function of rats following spinal cord contusion injury.

## Results

### Characterization of PLGA microspheres and their sustained release of factors *in vitro* and *in vivo*

**Figure 1:**
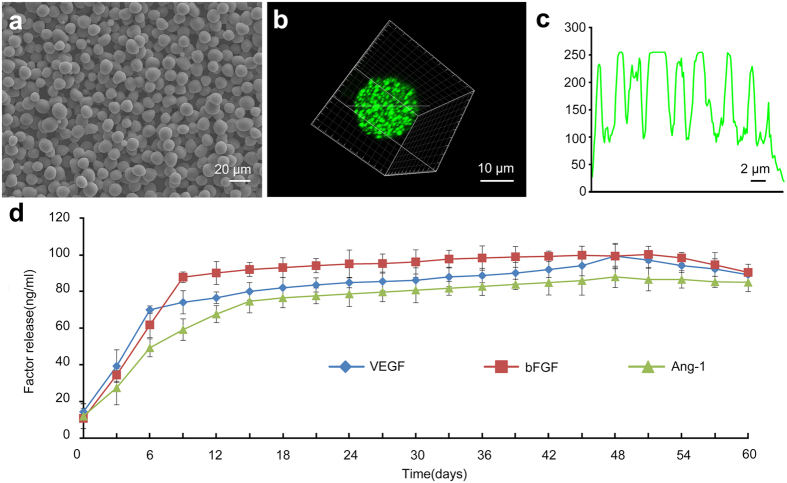
Characteristics of PLGA microspheres (PLGA-MS). Microspheres were prepared as described in Methods. (**a**) Representative scanning electron micrographs of PLGA-MS, showing spherical microspheres with smooth surfaces devoid of irregularities. Scale bar = 20 μm. (**b**) Representative confocal micrographs of a microsphere containing FITC-BSA. Scale bar = 10 μm. (**c**) The fluorescence intensity profile was analyzed along a line running through the center of the FITC-BSA microsphere. Scale bar = 2 μm. (**d**) *In vitro* release profiles of microspheres loaded with VEGF, Ang-1, or bFGF at 37 °C in PBS (pH 7.4). Data are shown as the mean ± standard deviation (n = 3).

**Figure 2:**
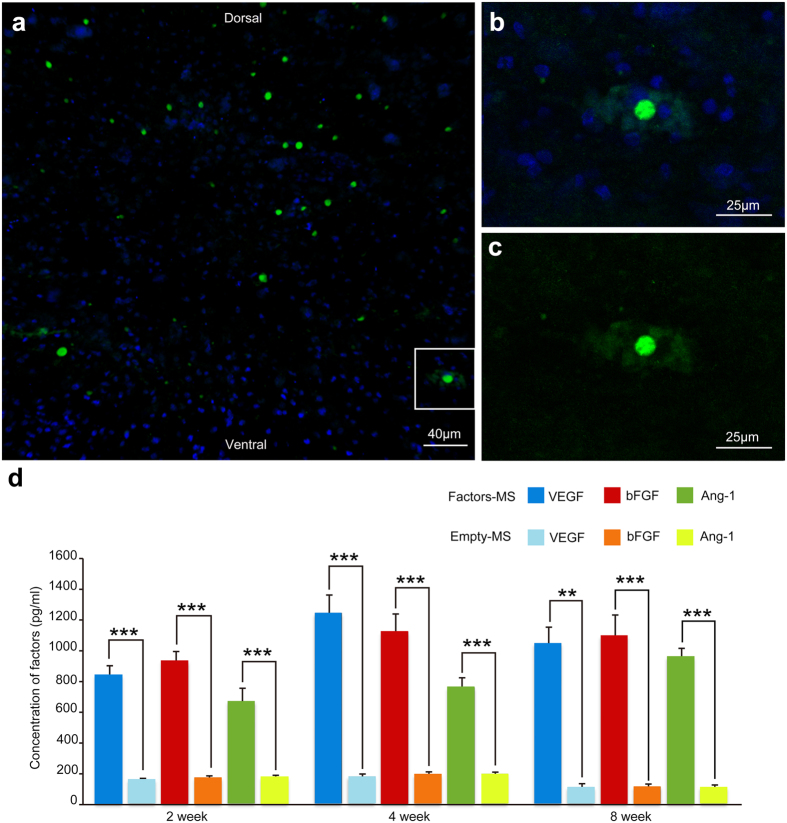
Release of different angiogenic factors from biodegradable PLGA microspheres *in vivo*. (**a**) Distribution of FITC-BSA microspheres at 3 days after injection into the injured spinal cord of rat. (**b**,**c**) Higher magnification of the area in the rectangle in (**a**). (**b**) Microspheres (green) and nuclei (blue) in spinal cord tissue. (**c**) Release and diffusion of FITC-BSA from PLGA microspheres into tissue. (**d**) Histogram showing *in vivo* release of angiogenic factors from microspheres into the spinal cord at 2, 4, and 8 weeks after spinal cord injury, based on ELISA of extracted tissues. Data are expressed as mean ± standard deviation (n = 4 for each group). Scale bar = 40 μm in (**a**) or 25 μm in (**b,c**).

Scanning electron microscopy showed the prepared microspheres to be spherical with a smooth surface (Fig. 1a), with a mean size of 9.74 ± 1.25 μm for angiogenic microspheres and 8.66 ± 1.65 μm for empty microspheres (*P* > 0.05). The encapsulation efficiency of PLGA microspheres was 83.4 ± 2.5%. The fluorescence intensity profile of FITC-BSA along the diameter of PLGA microspheres is indicated in Fig. 1b,c. *In vitro* release assays revealed that VEGF, Ang-1, and bFGF were released from the microspheres in a biphasic pattern (Fig. 1d), with rate of release rapidly increasing during the first 6–10 days, then remaining nearly constant over a prolonged phase, and finally tapering off starting around day 48. The released factors remained biological activity even 2 months after *in vitro* release (Supplemental Fig. 1). Serial confocal microscopy revealed FITC-BSA microspheres had already released the contents around the epicenter of spinal cord injury at 3 days after injury (Fig. 2a).

### Angiogenic microspheres promote angiogenesis and recruitment of neural precursors to the site of spinal cord injury in rats

**Figure 3:**
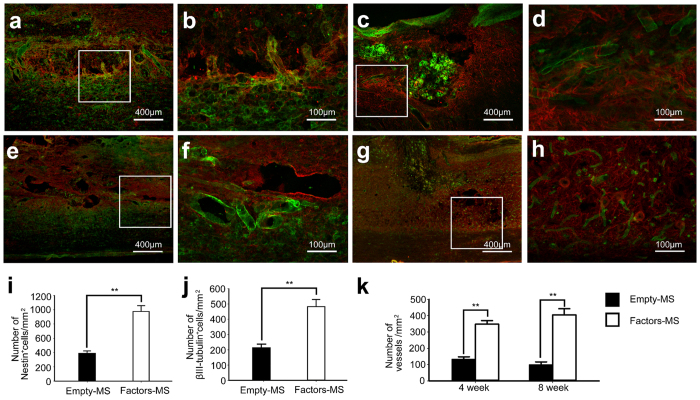
Angiogenesis and recruitment of endogenous neuronal precursors in injured spinal cord at 4 weeks after injection of angiogenic microspheres. (**a–d**) Representative photomicrographs of spinal cord sections after immunofluorescent labeling for nestin (red) and IB4-binding microvessels (green) in animals treated with (**a**) empty microspheres or (**c**) angiogenic microspheres. (**b,d**) Higher magnification of the area in rectangles in (**a**,**c**). Nestin-positive cells migrated into the injured spinal cord and localized within vessels in the lesion. (**e–h**) Representative photomicrographs of spinal cord sections after immunofluorescent labeling for βIII-tubulin (red) and IB4-binding microvessels (green) in animals treated with (**e**) empty microspheres or (**g**) angiogenic microspheres. (**f,h**) Higher magnification of the area in rectangles in (**e**–**g**). (**i**) Quantification of nestin-positive cells at the injury site; **P < 0.01 for the comparison of animals treated with empty or angiogenic microspheres. βIII-tubulin-positive cells migrated into the lesion and were closely associated with vessels. (**j**) Quantification of βIII-tubulin-positive cells at the injury site; **P < 0.01 for the comparison of animals treated with empty or angiogenic microspheres. (**k**) Quantification of IB4-binding vessels at the site of injury at 4 and 8 weeks after spinal cord injury; **P < 0.01 for the comparison of animals treated with empty or angiogenic microspheres. Data are expressed as mean ± SEM (n = 4 for each group). Scale bar = 400 μm in (**a,c,e,g**); or 100 μm in (**b,d,f,h**).

At 2–8 weeks following spinal cord injury, ELISA revealed that levels of VEGF, Ang-1, and bFGF in spinal cord tissues were significantly higher in animals treated with angiogenic microspheres than in animals treated with empty microspheres (Fig. 2d). Consistent with this result, the numbers of isolectin B4 (IB4)-binding vessles at the injury site at 4 and 8 weeks was significantly greater in animals treated with angiogenic microspheres than in control animals (*P* < 0.01; Fig. 3k). Animals treated with angiogenic microspheres also showed significantly greater numbers of cells positive for nestin or βIII-tubulin at the injury site (*P* < 0.01; Fig. 3a–j). Most of these cells resided with blood vessels (Fig. 3d,h). The findings indicate that the angiogenic microspheres effectively promoted angiogenesis and neural precursor recruitment to the injury site in our rat model. Cell proliferation at the injury site was observed using Ki67 immunofluorescence staining (Supplemental Fig. 2), confirming the contribution of functional vessels to spinal tissue regeneration.

### Angiogenic microspheres stimulate axonal growth after spinal cord injury in rats

**Figure 4:**
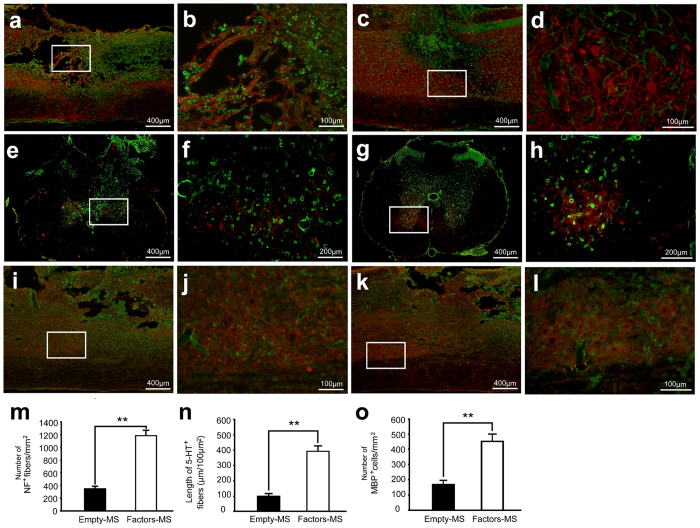
Angiogenesis with axonal growth and sprouting at the site of spinal cord injury at 8 weeks after injection of angiogenic microspheres. (**a–d**) Representative photomicrographs of spinal cord sections after immunofluorescent labeling for NF (red) and IB4-binding microvessels (green) in animals treated with (**a**) empty microspheres or (**c**) angiogenic microspheres. (**b**,**d**) Higher magnification of the area enclosed by the rectangles in (**a**,**c**). NF-positive fibers closely surrounded and aligned with vessels in the lesion, even traversing its epicenter. (**e–h**) Representative photomicrographs of spinal cord sections after immunofluorescent labeling for 5-HT (red) and IB4-binding microvessels (green) in animals treated with (**e**) empty microspheres or (**g**) angiogenic microspheres. (**f,h**) Higher magnification of the area enclosed by the rectangles in (**e**,**g**). 5-HT-positive fibers were observed around vessels in the ventral horn, 3–5 mm caudal to the lesion epicenter. (**i–l**) Representative photomicrographs of spinal cord sections after immunofluorescent labeling for MBP (red) and IB4-binding microvessels (green) in animals treated with (**i**) empty microspheres or (**k**) angiogenic microspheres. (**j–l**) Higher magnification of the area in rectangles in (**i**–**k**). MBP-positive cells in white matter were closely associated with vessels. (**m**) Quantification of NF-positive fibers at the injury site; **P < 0.01 for the comparison of animals treated with empty or angiogenic microspheres. (**n**) Quantification of sprouted 5-HT-positive fibers caudal to the lesion; **P < 0.01 for the comparison of animals treated with empty or angiogenic microspheres. (**o**) Quantification of MBP-positive cells adjacent to the lesion; **P<0.01 for the comparison of animals treated with empty or angiogenic microspheres. Data are expressed as mean ± SEM (n = 4 for each group). Scale bar = 400 μm in (**a**,**c**,**e**,**g**,**I**,**k**); or 100 μm in (**b**,**d**,**j**,**l**); or 200 μm in (**f**,**h**).

Next we examined whether increased angiogenesis and neural precursor recruitment to the site of spinal cord injury were associated with enhanced neural regeneration. Immunofluorescence microscopy revealed significantly greater density of neurofilament (NF)-positive fibers in the injured spinal cord of animals treated with angiogenic microspheres than in that of animals treated with empty microspheres (P < 0.01; Fig. 4a–d,m). At 8 weeks after spinal cord injury, analysis of tissues from animals treated with angiogenic microspheres revealed massive NF-positive fibers aligned with, and closely surrounding, blood vessels; fibers even occasionally traversed into the injury epicenter (Fig. 4d). In addition, serotonergic (5-HT) fibers were closely associated with blood vessels and were significantly longer than those in control tissues (*P* < 0.01; Fig. 4e–h,n). Numbers of MBP-positive mature oligodendrocytes were significantly higher in animals treated with angiogenic microspheres (*P* < 0.01; Fig. 4i–l,o), and these cells aggregated around blood vessels in the white matter region.

### Angiogenic microspheres are associated with significantly smaller loss of white matter volume

**Figure 5:**
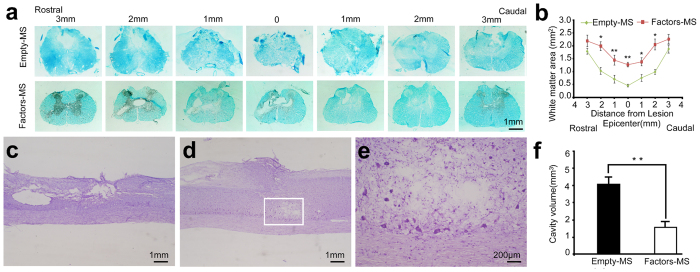
Reduction in lesion volume and white matter degeneration at 8 weeks after injection of angiogenic microspheres. (**a**) Representative photomicrographs of sections spanning from 3 mm rostral to 3 mm caudal to the lesion epicenter after staining with Luxol Fast Blue (LFB). (**b**) Quantification of LFB-positive spared white matter; *P < 0.05, **P < 0.01 for the comparison of animals treated with empty or angiogenic microspheres. (**c,d**) Sagittal sections stained with Nissl stain from animals treated with (**c**) empty microspheres or (**d**) angiogenic microspheres, showing cavity size in the contused spinal cord. (**e**) Higher magnification of the area in the rectangle in (**d**). (**f**) Quantification of cavity volume at the injury site; **P < 0.01 for the comparison of animals treated with empty or angiogenic microspheres. Data are expressed as mean ± SEM (n = 4 for each group). Scale bar = 1 mm in (**a**,**c**,**d**); or 200 μm in (**e**).

We examined myelination of the spinal cord 3 mm rostral and caudal to the epicenter of injury. Luxol fast blue staining of tissue from animals treated with angiogenic or empty microspheres showed that at 8 weeks after spinal cord injury, white matter volume progressively decreased toward the injury epicenter (Fig. 5a,b). However, white matter volume was significantly greater in animals treated with angiogenic microspheres (*P* < 0.01). Consistent with this result, these animals also showed significantly smaller cavity volume in the spinal cord (based on Nissl staining) than control animals (*P* < 0.01; Fig. 5c–f).

**Figure 6:**
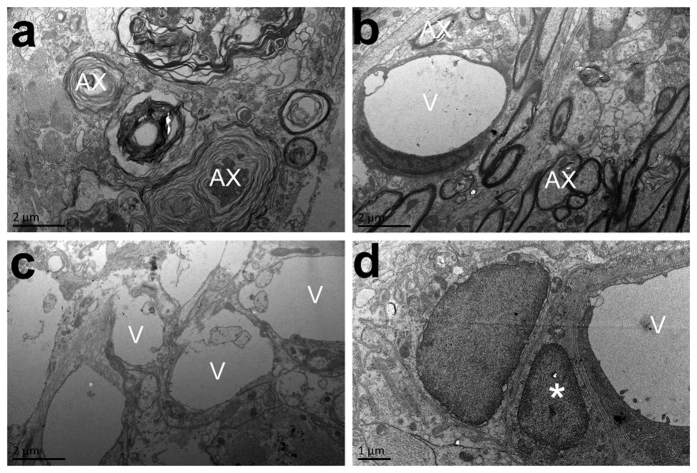
Transmission electron micrographs of the injury site at 12 weeks after injection of empty or angiogenic microspheres. (**a**) Abundant myelinated axons with loose myelin sheaths (Ax) were observed in the lesion area in the control group. (**b**) In contrast to the control group, the group treated with angiogenic microspheres showed abundant myelinated axons with relatively compact myelin sheaths (Ax) in the lesion; many of these axons were associated with blood vessels (V). (**c**) Tissue from animals treated with angiogenic microspheres also showed a microvascular network in the lesion area. (**d**) Certain cytoplasmic extensions from astrocytes (asterisks) made contact with adjacent blood vessels and other cells, based on criteria described in Methods. Scale bar = 2 μm in (**a**,**b**,**c**); or 1 μm in (**d**).

Electron microscopy of tissues taken at 12 weeks after spinal cord injury showed that in animals treated with angiogenic microspheres, most axons were myelinated and many followed blood vessels (Fig. 6b). In contrast, most axons in tissue from control animals were demyelinated (Fig. 6a). In tissues from animals treated with angiogenic microspheres, blood vessels were abundant at the injection site and lay adjacent to the lesion, even forming a microvascular network (Fig. 6c). Some astrocytes had cytoplasmic extensions making simultaneous contact with adjacent blood vessels and other cells (Fig. 6d).

### Angiogenic microspheres promote faster neurologic recovery

**Figure 7:**
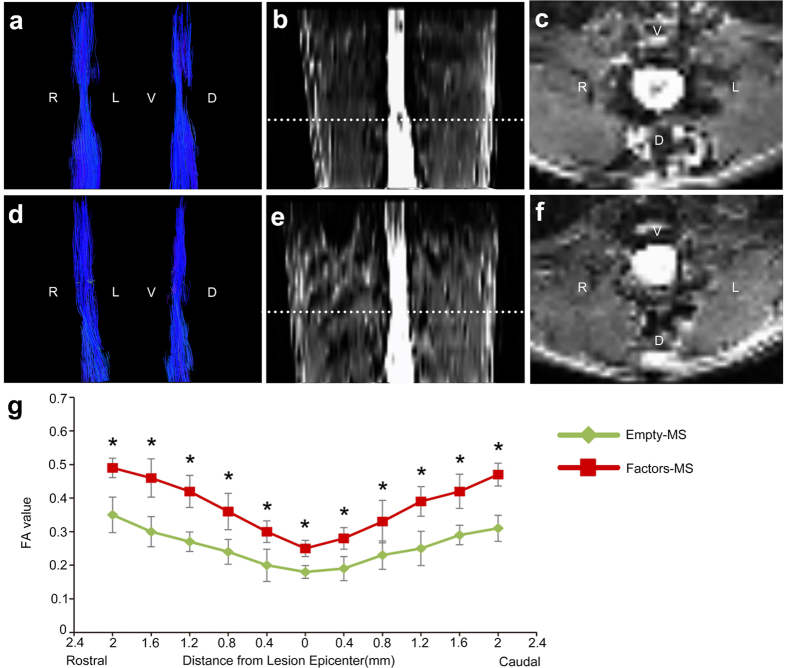
Tractography images and fractional anisotropy (FA) map of the spinal cord of rats at 12 weeks after injury. (**a**) Tractography image of the spinal cord in control animals treated with empty microspheres, showing the fracture of spinal fibers. (**b,c**) Coronal and transverse FA images of the injury epicenter in control animals. (**d**) Tractography image of the spinal cord in animals treated with angiogenic microspheres, showing more fibers in continuity. (**e**,**f**) Coronal and transverse FA images of the injury epicenter in animals treated with angiogenic microspheres. (**g**) Comparison of FA values along the spinal cord revealed significant differences between animals treated with empty or angiogenic microspheres at all locations. *P < 0.05 for the comparison of animals treated with empty or angiogenic microspheres. Data are expressed as mean ± SEM (n = 4 for each group). *R*, right; *L*, left; *V*, ventral; *D*, dorsal.

**Figure 8:**
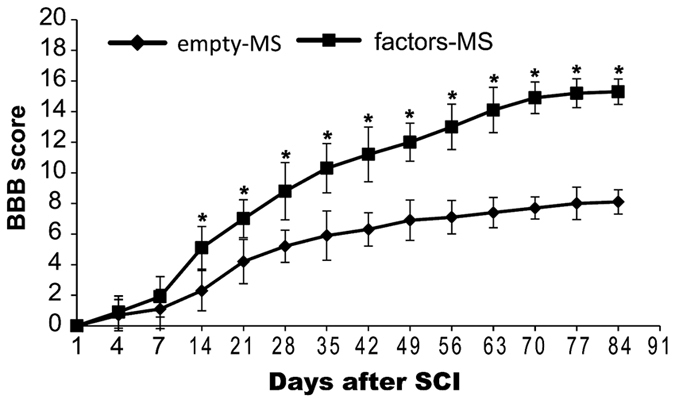
Microvasculature reconstruction promotes functional recovery. Mean Basso-Beattie-Bresnahan scores over 12 weeks after spinal cord injury and microsphere injection are shown. *P < 0.05 for the comparison of animals treated with empty or angiogenic microspheres. Data are expressed as mean ± SEM (n ≥ 10 for each group).

Tractography showed that at 12 weeks after spinal cord injury, significantly more fibers were continuous and travelled through the injury epicenter in animals treated with angiogenic microspheres than in control animals (Fig. 7a,d; Supplementary Videos). Fractional anisotropy (FA) imaging of the spinal cord (Fig. 7b,c,e,f) showed that at 8 weeks after injury, FA in both animal groups increased with increasing distance from the injury epicenter (Fig. 7g), and it was significantly higher in animals treated with angiogenic microspheres (*P* < 0.01). Open field tests showed that spinal cord injury resulted in complete paralysis, followed by gradual recovery in both groups. Recovery was significantly greater in animals that received angiogenic microspheres (BBB score at 12 weeks, 15.3 ± 0.83) than in those that received empty microspheres (BBB score at 10 weeks, 7.7 ± 0.72) (*P* < 0.01; Fig. 8).

### Angiogenic microspheres stimulate miR-210 expression in spinal cord tissues

**Figure 9:**
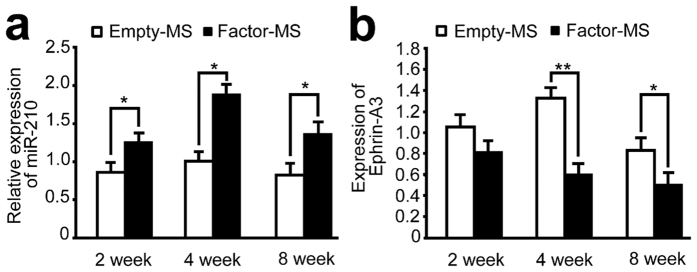
Angiogenic microspheres up-regulate miR-210 expression and down-regulate ephrin-A3 expression in spinal cord tissues. (**a**) Time course of expression of endogenous miR-210 after spinal cord injury and microsphere injection, based on real-time PCR. Results were normalized to those for SnRNAU6. (**b**) Expression of ephrin-A3 based on real-time PCR, showing down-regulation concomitant with up-regulation of miR-210 in animals treated with angiogenic microspheres at 4 and 8 weeks after injury [see panel (**a**)]. *P < 0.05, **P < 0.01 for the comparison of animals treated with empty or angiogenic microspheres. Data are expressed as mean ± SEM (n = 4 for each group).

Local administration of miR-210 at sites of spinal cord injury in rats knocks down ephrin-A3 expression, promoting angiogenesis^20^. Therefore we wanted to examine whether the enhanced neural regeneration observed in our animals treated with angiogenic microspheres was associated with changes in expression of miR-210 and ephrin-A3. Real-time RT-PCR assays at 2, 4, and 8 weeks after injury revealed significantly higher levels of endogenous miR-210 in injured spinal cord tissues in animals treated with angiogenic microspheres than in control tissue (*P* < 0.01; Fig. 9a). Consistent with this result, levels of endogenous ephrin-A3 mRNA at 4 and 8 weeks after injury were significantly lower in tissues from animals treated with angiogenic microspheres than in control tissues (*P* < 0.01; Fig. 9b).

## Discussion

This study used the electrospray technique to encapsulate angiogenic factors in biodegradable PLGA microspheres. Compared to the technique of water-in-oil-in-water (W_1_/O/W_2_) double emulsion solvent evaporation, the electrospray technique can increase encapsulation efficiency without causing protein denaturation during microencapsulation^21^,^22^,^23^. Using this technique, we achieved a mean particle size of approximately 9.7 μm and an encapsulation efficiency (83.4%) substantially higher than that usually obtained by double emulsion solvent evaporation^24^. We demonstrate that sustained co-delivery of VEGF, Ang-1 and bFGF from PLGA microspheres at the epicenter of spinal cord injury stimulates axonal growth after spinal cord injury in rats, which is associated with enhanced angiogenesis and recruitment of neural precursors to the site of injury. Co-delivery of these angiogenic factors is also associated with faster recovery of neurologic function. These findings suggest that co-delivery of angiogenic factors via sustained release from PLGA microspheres may effectively promote neural regeneration and recovery of neurologic function following spinal cord injury.

A favorable vascular microenvironment is crucial for recovery from spinal cord injury^2^. After contusive spinal cord injury, microvascular dysfunction and endothelial cell loss occur, followed by formation of new blood vessels at the epicenter and in the penumbra^25^. Although this endogenous angiogenic response may help improve the temporal vascular microenvironment^26^, sustained angiogenic response is required for recovery from spinal cord injury by sparing neural tissue from further damage and by triggering regeneration of new tissue. We have previously demonstrated that co-delivery of VEGF and Ang-1 effectively promotes local angiogenesis in a mouse stroke model^18^. The current study added a third angiogenic factor, bFGF, which is known to promote neural regeneration and recovery of neurologic function following spinal cord injury^11^,^12^. Here we showed that sustained release of angiogenic factors from PLGA microspheres facilitated neurite regrowth and angiogenesis, increasing blood vessel density at the site of spinal cord injury. Our findings are consistent with those of other studies showing that concurrent use of multiple angiogenic proteins enhances angiogenesis^27^,^28^, making axonal regeneration more likely.

Development of new capillary sprouts in tissues requires dynamic, spatiotemporally regulated interaction among different factors, including angiogenic factors, endothelial cells, and surrounding matrix proteins^28^. When using angiogenic factors encapsulated into microspheres to promote revascularization, the sequence and rate of drug release should be considered. Controlled release was achieved in the current study by using PLGA of different molecular weights, because PLGA biodegradation slows down with increasing molecular weight^18^,^29^. Our results in the present study on the release and diffusion of FITC-BSA from microspheres at the site of injury are consistent with the suggestion by Loy *et al*.^17^ that the window for angiogenic therapy falls within the subacute period of spinal cord injury (3–7 days after injury). We used FITC-conjugated Griffonia simplicifolia IB4 to track microvasculature remodelling at the injury site and determine the perfusion status of functional vessels that were anastomosed with host vessels, similar to previous studies^30^,^31^. We observed an increase in the density of functional vessels at the injury epicenter as early as 4 weeks after spinal cord injury. In contrast to a previous study^26^, we failed to observe any vessel regression, suggesting that our controlled, sustained release of angiogenic factors from PLGA microspheres provides a promising platform for angiogenic therapy following spinal cord injury. The platform may also be useful for other *in vivo* applications.

Endogenous neural stem/progenitor cells (eNSPCs) have been found in the ependymal regions lining the central canal in the spinal cord^32^. Spinal cord injury can initiate an endogenous neurogenesis response where eNSPCs proliferate and migrate from the region of the central canal, differentiating into oligodendrocytes, astrocytes, and neurons, which play an important role in reorganization after spinal cord injury^33^,^34^. Several therapeutic strategies to activate eNSPCs have been investigated in spinal cord injury because eNSPCs avoid the problems of immune rejection, tumor formation, and ethical concerns, which are significant challenges for exogenous stem cell transplantation^35^. A close association of angiogenesis with neurogenesis has been established in many studies^36^,^37^. Angiocrine factors from new blood vessels attract migrating immature neurons to the lesion site, promoting recovery of injured tissue after stroke^3^,^4^. Consistently, we show in the current study that enhanced angiogenesis is associated with increased neurogenesis at the lesion site. Notably, we observed increased recruitment of neural progenitors to the lesion site and these progenitors were closely associated with blood vessels. The microvascular network may provide an effective “bridge” across the lesion cavity and support and guide axonal climbing or growth. We found that many serotonergic axons descended below the level of the lesion in animals treated with angiogenic microspheres at 8 weeks after spinal cord injury, which may contribute to recovery of motor function^38^. Our tractography further demonstrated regeneration of damaged nerve fibers beyond the injury epicenter at 12 weeks after spinal injury. We also observed a significantly spared rim of white matter, including at the lesion epicenter, and most MBP-positive cells were found around vessels near the lesion site. These MBP-positive cells may promote re-myelination, helping to shrink the area of parenchymal damage after spinal cord injury. FA, which serves as an index of microstructural changes and physiological state of injured nerve fibers, was significantly greater in animals receiving angiogenic microspheres. Many myelinated axons were seen in the lesion area in electron micrographs of negatively stained tissue sections and microscopy of Luxol fast blue-stained tissue sections. These findings together suggest that angiogenic microspheres stimulate angiogenesis at the site of spinal cord injury, helping enhance neural regeneration in rats.

The miR-210 is implicated in the regulation of many cellular processes, including cell cycle, development, and membrane trafficking^39^,^40^. Ujigo *et al*. reported that miR-210 was upregulated at the injury site after spinal cord injury and promoted angiogenesis by suppressing ephrin-A3 expression^20^. We observed that miR-210 levels at the lesion site were significantly higher in animals that received angiogenic microspheres than in those that received empty microspheres. As expected, animals receiving angiogenic microscopheres also showed significantly lower expression of ephrin-A3. Ephrin-A3 is an endogenous negative modulator of neurogenesis in the adult CNS, regulating neurogenesis from neural progenitors in diverse CNS regions^41^. In the present study, angiogenesis induced by angiogenic factors reduced ephrin-A3 expression at the injury site. This may promote migration and proliferation of eNSPCs, thereby enhancing endogenous regeneration of spinal tissues. Indeed, our microspheres loaded with VEGF, Ang-1, and bFGF may promote regeneration via two parallel pathways. One pathway is a positive feedback loop in which angiogenesis activated by the three factors promotes miR-210 expression, which induces endogenous VEGF production, leading to further angiogenesis. At the same time, miR-210 up-regulation directly suppresses ephrin-A3 expression, stimulating neurogenesis at the injury site. Of course, the gene regulation mechanism of neurogenesis associated with angiogenesis needs to be further elucidated.

## Methods

### Microsphere preparation and characterization

Microspheres of PLGA [50:50, lactic/glycolic (%); Sigma, St Louis, MO, USA] were prepared aseptically using a modified electrospray technique^21^. Bovine serum albumin (10 mg; BSA; Sigma) or fluorescein isothiocyanate-BSA (FITC-BSA, Sigma) in 0.10 mL deionized water were dispersed into the organic phase (0.12 g PLGA in 2 mL dichloromethane) by sonication for 5 s at 300 Hz for 5 times at an interval of 10 s. The resulting water-in-oil emulsion was electrosprayed onto aluminum foil (collector plate) at a flow rate of 1 mL/h using a syringe pump. A high voltage of 6 kV was applied between the spinneret (27-G needle) and the collector plate, located 20 cm from the spinneret. The working conditions were optimized to ensure continuous thin jet flow. For preparation of angiogenic microspheres, 10 μg VEGF (R&D Systems, Minneapolis, MN, USA) was dissolved in 25 μL of 0.1% (w/v) BSA, then added to 0.5 mL of 6% (w/v) PLGA (50:50, Mw 7–17 kDa). Ang-1 or bFGF (25 μg; R&D Systems) was dissolved in 50 μL of 0.1% (w/v) BSA, then added to 1 mL of 6% (w/v) PLGA (50:50; Mw 38–54 kDa for Ang-1, Mw 24–38 kDa for bFGF). A water-in-oil emulsion was formed by sonication and then electrosprayed. After solvent evaporation, the microspheres were harvested from the aluminum foil, freeze-dried and stored at −20 °C. For preparation of empty microspheres, 1 mL of 6% (w/v) PLGA solution (50:50, Mw 24–38 kDa) was electrosprayed using the same method as for angiogenic microspheres but in the absence of angiogenic factors or BSA.

Microsphere morphology was examined using scanning electronic microscopy (SEM; 6460 LV, JEOL, Tokyo, Japan). Microspheres were mounted onto metal stubs using double-sided adhesive tape. They were vacuum-coated with a thin layer of platinum and then examined by SEM at 15 kV. The diameter of microspheres was measured and averaged using Image-Pro Plus 6.0 software (Media Cybernetics, Silver Spring, MD, USA). The fluorescence intensity profile was determined from confocal microscopy images using Image-Pro Plus. Encapsulation was confirmed by laser scanning confocal microscopy (LSM 780, ZEISS, Germany). Encapsulation efficiency was calculated as the ratio of the actual to theoretical amount of BSA encapsulated. Briefly, 10 mg dried PLGA microspheres containing BSA were dissolved in 0.5 mL acetonitrile and mixed thoroughly at ambient temperature for 24 h. BSA concentration was then determined using a P-Class NanoPhotometer^®^ (P 330, Implen, Munich, Germany) in quadruplicate.

### Animals and overall study design

All procedures were performed according to the US National Institutes of Heath Guide for the Care and Use of Laboratory Animals. The study protocol was approved by the Institutional Animal Care and Use Committee of Capital Medical University, Beijing, China. Seventy adult female Sprague–Dawley rats (250–280 g) were housed at 23 ± 1 °C with a humidity of 50–60% and a 12-h light/dark cycle. Animals had free access to a standard diet and water.

Two rats were injected with FITC-BSA microspheres, and effects of protein release from microspheres were analyzed *in vivo* 3 days later. The remaining rats were randomly divided into a group that received empty microspheres (n = 34) and a group that received angiogenic microspheres (n = 34). Twelve rats from each group were analyzed for histomorphology, mainly by immunohistochemistry: at 4 weeks after microsphere injection, tissue longitudinal sections from 4 rats in each group were stained for nestin or βIII-tubulin; at 8 weeks after microsphere injection, tissue longitudinal sections from 4 rats in each group were stained for NF, MBP, or Nissl stain; at 8 weeks after microsphere injection, tissue transverse sections from 4 rats in each group were stained for 5-HT or Luxol fast blue stain. At 2, 4 and 8 weeks after microsphere injection, tissues from 4 rats in each group (per time point) were analyzed by ELISA and real-time PCR to measure changes in VEGF, Ang-1 and bFGF as well as in endogenous expression of miR-210 and ephrin-A3. In the final phase of the study, the remaining 10 rats were evaluated for BBB scores, of which 4 animals were randomly selected for diffusion tensor imaging (DTI) followed by ultrastructural analysis at 12 weeks after microsphere injection.

### Establishment of a rat model of spinal cord injury

Rats (n = 34 per group) were randomized to receive empty microspheres (control) or angiogenic microspheres. Rats were anesthetized with intraperitoneal 6% chloral hydrate (6 mL/kg), and the spinal column was exposed at T8–T10. Laminectomy at T9 was performed and injury was induced using the Impact One™ Stereotaxic Impactor (Leica, Wetzlar, Germany)^42^. The following mechanical parameters were used^43^: impactor tip size, 1.5 mm; impact velocity, 1.5 m/sec; cord displacement, 1.7 mm; dwell time, 100 ms. Immediately after injury, angiogenic microspheres containing a mixture of 5 μL each of VEGF, Ang-1, and bFGF or empty microspheres (15 μL) were stereotaxically injected at a controlled speed of 1 μL/min into the epicenter of the lesion using a 25-μL Hamilton syringe fitted with a glass micropipette. The needle was kept in place for an additional 10 min before being slowly retracted.

### Angiogenic factor release assays *in vitro* and *in vivo*

*In vitro* release of VEGF, Ang-1, or bFGF from PLGA microspheres was analyzed by incubating 5 mg microspheres in 1 mL PBS (pH 7.4) at 37 °C with rotation at 25 rpm. At defined times, release medium with PLGA microspheres was clarified by centrifugation (20,000 g, 15 min), and 1 mL supernatant was taken to store at −80 °C. At the same time, 1 mL fresh PBS was added to mix microspheres and keep the volume of release medium constant. Levels of VEGF, Ang-1, and bFGF in the release medium were assayed using commercial ELISA kits (R&D Systems).

At 3 days after spinal cord injury and injection of FITC-BSA microspheres, 2 rats were perfused with 0.01 mM PBS (pH 7.4, 37 °C), followed by 4% (w/v) paraformaldehyde in 0.1 M PBS (pH 7.4, 4 °C). Tissue blocks of the spinal cord were cut serially into 20-μm longitudinal sections. *In vivo* release of FITC-BSA was observed at the injection site using a laser scanning confocal microscopy (LSM 780, ZEISS, Germany).

*In vivo* release of angiogenic factors was observed as follows. At 2, 4 and 8 weeks after spinal cord injury and injection of empty or angiogenic microspheres, rats were perfused with 0.01 mM PBS (pH 7.4, 37 °C). Spinal cord tissue encompassing the injury site was dissected and cut into a segment of approximately 10 mm long. Each tissue block was divided into two equal parts along its longitudinal median: one part was analyzed using ELISA to measure release of angiogenic factors; the other part was analyzed using real-time PCR to detect changes in expression of miR-210 and ephrin-A3. Fresh spinal cord tissue was lysed using lysis buffer [50 mM Tris-Cl, 2% (w/v) sodium dodecyl sulfate (SDS), 10% (v/v) glycerol, pH 7.4] and sonicated. After centrifugation at 12500 g for 30 min, the supernatant was collected and analyzed by ELISA kits (R&D Systems) to determine levels of VEGF, Ang-1, and bFGF. Optical density was determined at 450 nm using a microplate reader.

### Immunohistochemistry

Rats were first injected with 250 μL of 2 mg/mL FITC-conjugated Griffonia simplicifolia isolectin B4 (IB4; Sigma, St, Louis, MO, USA) through the right external jugular vein, and the drug was allowed to circulate for 25 min prior to perfusion^30^,^44^. Then animals were perfused with 0.01 mM PBS (pH 7.4, 37 °C), followed by 4% (w/v) paraformaldehyde in 0.1 M PBS (pH 7.4, 4 °C). Spinal cord tissue blocks were embedded in Optimal Cutting Temperature compound and cut serially into 20-μm longitudinal sections and 20-μm transverse sections on a cryostat microtome (Leica CM 3500, Wetzlar, Germany). All sections were thawed and mounted on gelatin-coated glass slides. The following primary antibodies were used (Abcam, Cambridge, UK): rabbit anti-βIII-tubulin (1:500), rabbit anti-MBP (1:200), rabbit anti-serotonin transporter (5-HT, 1:500), mouse anti-nestin (1:200), and mouse anti-neurofilament medium (NF-09, 1:500). Secondary antibodies (1:500; Invitrogen, Carlsbad, CA, USA) included goat anti-rabbit Alexa Fluor 594 and goat anti-mouse Alexa Fluor 594. Immunofluorescence was visualized under a fluorescence microscope (IX 73, Olympus, Center Valley, PA, USA).

IB4 binding blood vessels were quantitatively assessed using Image Pro Plus software^44^,^45^,^46^. Of the 20-μm longitudinal sections spanning the entire thickness of the spinal cord, every 10th section was sampled. The lesion area in each section was defined as areas exhibiting disorganization of spinal tissue and/or areas within the lesion cavity. Lesion areas were manually outlined for each section (10–12 sections/experimental case), and the total area was automatically calculated by the software. Only tube-like structures with green labelling were considered blood vessels; these vessels were counted manually at 200× magnification, and the per-area density of blood vessels was calculated for each image by dividing the total number of blood vessels by the area. Four rats from each group were examined at each time point. In serial longitudinal sections 200 μm apart, cells in lesion areas that were positive for βIII-tubulin or nestin and that had a DAPI-labelled nucleus, NF-positive axons in lesion areas, and cells in regions adjacent to the lesion that were positive for MBP and that had a DAPI-labelled nucleus were counted using the same method as for IB4 binding blood vessels (n = 4 for each group at 4 or 8 weeks after injury). Mean counts for each treatment and time point were reported. Furthermore, 5-HT-positive fibers in the ventral horn of serial cross-sections 200 μm apart (n = 4 for each group at 8 weeks after injury) and located 3–5 mm caudal to the lesion epicenter were traced using Image Pro Plus. Cumulative length of fiber per area was then determined^47^. The values from each section were averaged, and mean cumulative length was calculated for each treatment group. All measurements and counts were made by an observer blinded to experimental conditions.

### Luxol fast blue and Nissl staining

White matter in spinal cord tissues was stained using Luxol fast blue as described^44^ and quantified using Image Pro Plus. Luxol fast blue-stained tissue sections located the lesion epicenter, 1, 2 and 3 mm rostral and caudal to the lesion epicenter were analyzed. Furthermore, the lesion cavity in longitudinal sections of the spinal cord was visualized by Nissl staining and quantified as described^45^. Lesion regions were outlined using Image Pro Plus, and cavity volumes were determined using the following equation: V = Σ [area × section thickness × number of sections in each sampling block].

### Diffusion tensor imaging (DTI)

DTI datasets were obtained at 12 weeks after injury and injection of angiogenic or empty microspheres. Animals (n = 4 per group) were anesthetized with intraperitonal 6% chloral hydrate (6 mL/kg). All data were acquired on a 7.0-T BrukerBioClinSpin Animal MRI System (Bruker, Billerica, MA) using a specialized coil for scanning rats. The rat was placed in the coil in a prone position. Data were acquired in the region of spinal cord contusion. DTI experiments were performed using the sequence: single-shot spin-echo echo-planar imaging (SE-EPI). Imaging parameters were as follows: field of view (FOV), 36 × 30 mm; acquisition matrix, 128 × 128; slice thickness, 0.8 mm; slice number, 25; inter-slice gap/slice thickness, 30%; echo time (TE), 5000 ms; repetition time (TR), 60 ms. Diffusion encoding was in 20 various non-collinear and non-coplanar diffusion directions with a b-value of 600 s/mm^2^.

### Transmission electron microscopy

Animals analyzed by DTI were sacrificed, perfused with 0.01 mM PBS (pH 7.4, 37 °C), followed by 4% (w/v) paraformaldehyde in 0.1 M phosphate buffer (pH 7.4, 4 °C) plus 2% glutaraldehyde. Tissue blocks (1 mm^3^) from the epicenter of injured tissue were postfixed in 3% glutaraldehyde for 2 h at room temperature, and the tissue was embedded in Epon 812. Ultrathin sections were cut with an ultramicrotome (Leica RM 2165, Leica Ultracut UCT, Germany) and stained with uranyl acetate and lead citrate. Observations and photomicrographs were made using a transmission electron microscope (TEM; JEOL JEM-2100, Japan) operated at 60 kV. Cells were identified as astrocytes if they satisfied all of the following morphological criteria: (1) small, polygonal cell body with distinct oval or elongated nucleus; (2) long cell processes covering nearly 80% of the abluminal side of the basement membrane of capillary vessels, which consists of endothelial cells, pericytes and basement membrane; and (3) presence of mitochondria in cell processes associated with capillary vessels.

### Tractography and fractional anisotropy (FA)

Tensor reconstruction and fiber tractography were performed using Diffusion Toolkit v0.6 (http://www.trackvis.org). FA maps, three eigenvalues (λ_1_, λ_2_, λ_3_) and their corresponding eigenvectors were acquired. Tracts were visualized and DTI parameters were quantified using TrackVis v0.5 (Harvard Medical School, Boston, USA). The injury epicenter of the spinal cord was established at T9 on the FA map. A 2-mm segment was then measured from the epicenter both rostrally and caudally and further subdivided into 0.4-mm increments. Regions of interest (ROIs) were defined, and FA values were generated by outlining the entire cord parenchyma in the axial plane of FA maps. Average FA within each group was plotted for different locations along the spinal cord.

### Open-field test

Hindlimb motor function was evaluated in open-field tests using the Basso-Beattie-Bresnahan (BBB) scale at 1, 4, and 7 days after spinal cord injury, and weekly thereafter. Rats were placed on a flat surface with a diameter of 1 m and observed for 3 min. A score of 0 represents flaccid paralysis; 21, normal gait. Two blinded observers simultaneously evaluated animal behavior.

### Real-time PCR

Total cellular RNA was isolated using TRIzol (Invitrogen) according to the manufacturer’s instructions. First-strand cDNA synthesis was carried out using the TaqMan MicroRNA Reverse Transcription Kit (Applied Biosystems, Foster City, CA), and the Ready-To-Go You-Prime First-Strand Beads (GE Healthcare, UK) with oligo(dT) primers. Real-time polymerase chain reaction (PCR) was performed using TaqMan microRNA assay kits (Applied Biosystems) for mature miR-210 and its target gene, ephrin-A3 (Efna3; XM_574979). For normalization, parallel reactions were carried out targeting the genes SnRNAU6 or GAPDH (ACTB; Hs99999905_m1). Cycle thresholds were determined from the exponential phase of amplification, and expression levels were quantified relative to the normalization controls using standard curves.

### Statistical analysis

Data were expressed as mean ± SD or SEM and analyzed using SPSS 19.0 (IBM, Armonk, NY, USA). Differences in histological data, including cell counts, vessel counts, axon counts, axon length, and cavity volume, were assessed for significance using Student's t test. Differences in BBB scores, white matter volume, or FA values were assessed using two-way analysis of variance (ANOVA) with repeated measures, followed by the post-hoc Bonferroni test. Differences in real-time PCR results and *in vivo* release profiles were assessed using one-way ANOVA followed by the post-hoc Scheffe test. The significance level for all tests was set at *P* < 0.05.

## Conclusions

We have encapsulated three angiogenic factors into biodegradable, biocompatible PLGA microspheres and achieved the sustained release of these factors at the injury epicenter *in vivo*. These angiogenic microspheres stimulated angiogenesis and promoted neurogenesis in rats with spinal cord injury, accelerating recovery of neurologic function. This study demonstrates the feasibility and efficacy of this minimally invasive therapeutic approach, and encourages further study of its application in situations requiring controlled, sustained drug delivery.

## Additional Information

**How to cite this article**: Yu, S. *et al*. Angiogenic microspheres promote neural regeneration and motor function recovery after spinal cord injury in rats. *Sci. Rep.* **6**, 33428; doi: 10.1038/srep33428 (2016).

## Supplementary Material

Supplementary Videos S1

Supplementary Videos S2

Supplementary Information
