# Supplementary material for: Angiogenic microspheres promote neural regeneration and motor function recovery after spinal cord injury in rats
Source: Sci Rep. 2016 Sep 19;6:33428. doi: 10.1038/srep33428 (PMC5027575; doi:10.1038/srep33428)
Supplement: Supplementary Information [file srep33428-s3.doc]

Angiogenic microspheres promote neural regeneration and motor function recovery after spinal cord injury in rats

Shukui Yu,^a^ Shenglian Yao,^b,c^ Yujun Wen,^a,d^ Ying Wang,^e^ Hao Wang^e^ and Qunyuan Xu*^a^

^a^Department of Neurobiology, Beijing Institute for Brain Disorders, Beijing Center of Neural Regeneration and Repair, Beijing Key Laboratory of Major Brain Disorders, Capital Medical University, 10 Xitoutiao, You An Men, Beijing 100069, China.

^b^School of Materials Science and Engineering, University of Science and Technology Beijing, 30 Xueyuan Road, Beijing 100083, China

^c^School of Materials Science and Engineering, Tsinghua University, Hai Dian, Beijing 100084, China

^d^Ningxia Key Laboratory of Cerebrocranial Diseases, Department of Anatomy, Histology and Embryology, School of Basic Medical Sciences, Ningxia Medical University, 1160 Shengli Street, Yinchuan 750004, China

^e^Department of Anatomy, School of Basic Medical Sciences, Capital Medical University, 10 Xitoutiao, You An Men, Beijing 100069, China

*Correspondence author: Qunyuan Xu

E-mail: xuqy@ccmu.edu.cn

Fax: +86010-83911464，Tel: +8613693636886

The biological activity of factors released from microspheres was assessed using cultures of endothelial progenitor cells (EPCs). Rat bone marrow was obtained by flushing tibiae and femurs of Sprague–Dawley rats (250–300 g), and mononuclear cells were isolated by density gradient centrifugation using Histopaque-1083 (Sigma-Aldrich, St. Louis, MO, USA). Mononuclear cells were seeded in 6-well plates pre-coated with fibronectin (R&D Systems, Minneapolis, MN, USA) at a density of 2.5 × 10^6^ cells/cm^2^. Cells were maintained in EGM-2MV (Lonza, Walkersville, MD, USA), which consists of EBM-2 basal medium, 5% fetal bovine serum (FBS) and SingleQuots (Lonza) containing vascular endothelial growth factor (VEGF), basic fibroblast growth factor (bFGF), insulin-like growth factor (IGF), endothelial growth factor (EGF), heparin, ascorbic acid, and the antibiotics gentamicin and amphotericin B. Nonadherent cells were discarded after 24 h, and the culture medium was replaced every 2–3 days. Approximately 10 days later, EPC colonies appeared that could be morphologically identified under the microscope.

These EPC cultures were incubated in three types of EGM-2MV, each supplemented with release medium containing released Ang-1, VEGF or bFGF that had been harvested on day 63 and assayed by ELISA. The three types of EGM-2MV contained (1) release medium with 10 ng/mL Ang-1, for which EGM-2MV without Ang-1 release medium served as a negative control; (2) release medium with 10 ng/mL VEGF instead of the VEGF in the SingleQuots in complete medium, for which EGM-2MV without VEGF served as a negative control; or (3) release medium with 10 ng/mL bFGF instead of the bFGF in the SingleQuots in complete medium, for which EGM-2MV without bFGF served as negative control. The EPC cultures were exposed to release medium of angiogenic microspheres as described for *in vivo* release assays. After 10 days, EPC colonies were visualized under a microscope (IX73, Olympus, Center Valley, PA, USA). Cells cultured in EGM-2MV without Ang-1 release medium formed typical cobblestone colonies (Fig. 1a). Cells cultured in EGM-2MV containing Ang-1 release medium formed colonies with lumen-like structures (Fig. 1c), similar to cells cultured in EGM-2MV containing Ang-1 factor (Fig. 1b). This indicates that Ang-1 released from microspheres was still active after 2 months. Similarly, cells cultured in EGM-2MV without VEGF or bFGF did not form typical cobblestone colonies (Fig. 1d and 1f), whereas cells cultured in EGM-2MV containing VEGF or bFGF release medium did (Fig. 1e and 1g), as did cells cultured in EGM-2MV containing VEGF or bFGF factor (Fig. 1a). These results indicate that VEGF and bFGF released from microspheres were still active after 2 months.


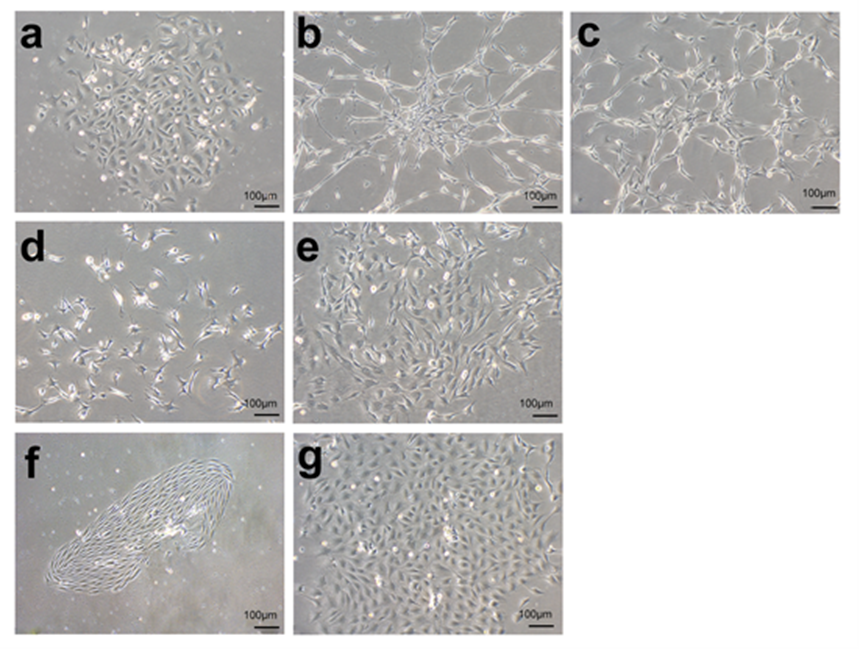


**Supplementary Fig. S1** Representative micrographs of EPC colonies from primary cells cultured for 10 days. (a) Cells cultured in EGM-2MV without Ang-1 release medium formed typical cobblestone colonies. (b) Cells cultured in EGM-2MV with Ang-1 factor formed lumen-like colonies. (c) Cells cultured in EGM-2MV containing Ang-1 release medium also formed lumen-like colonies. (d) Cells cultured in EGM-2MV without VEGF did not form colonies. (f) Cells cultured in EGM-2MV without bFGF did not form typical cobblestone colonies. (e and g) Cells cultured in EGM-2MV containing VEGF or bFGF release medium formed typical cobblestone colonies. Scale bar=100 μm.

To confirm that functional vessels contributed to the observed regeneration of spinal tissue, cell proliferation at the site of injury was assessed using Ki67 immunofluorescence staining. The primary antibody was rabbit anti-Ki67 (1:500, Abcam, Cambridge, UK), and secondary antibody was goat anti-rabbit Alexa Fluor 594 (1:500, Invitrogen, Carlsbad, CA, USA). Immunofluorescence was visualized using laser scanning confocal microscopy (LSM 780, ZEISS, Germany). At 4 weeks after spinal cord injury, abundant Ki67-positive cells were observed at the injury site in animals treated with angiogenic microspheres, most of which surrounded functional vessels (Fig. 2a to 2e); scant Ki67-positive cells were observed in animals treated with empty microspheres (Fig. 2f to 2j). By 8 weeks after injury, the numbers of Ki67-positive cells had decreased in animals treated with angiogenic microspheres, with the remaining cells still near blood vessels (Fig. 2k to 2o). These results suggest that functional vessels can initiate proliferation of neighboring cells, promoting regeneration of spinal tissue at the site of injury.


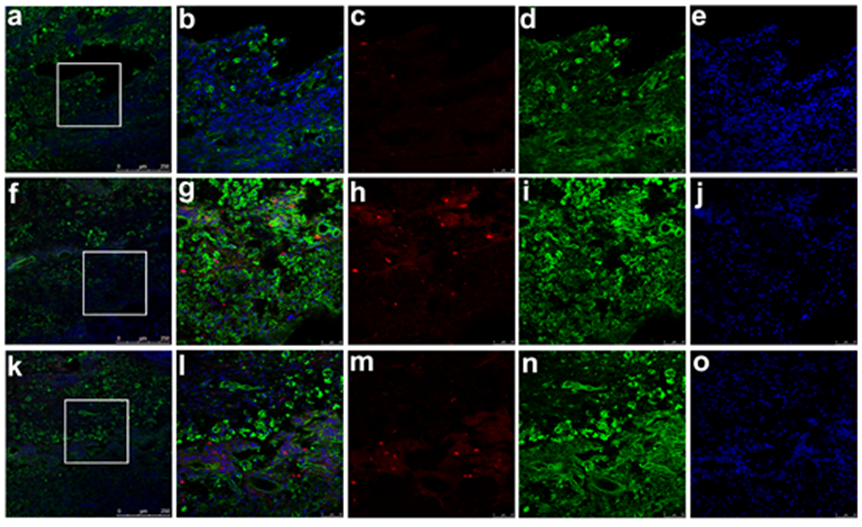


**Supplementary Fig. S2** Cell proliferation at the injury site following injection of angiogenic microspheres. Representative photomicrographs of spinal cord sections after immunofluorescent labeling for Ki67 (red), IB4-binding microvessels (green) and nuclei (DAPI, blue). (a-e) Results at 4 weeks after spinal cord injury in animals treated with empty microspheres. (b) Higher magnification of the area in the rectangle in (a). Scant Ki67-positive cells (c) and IB4-binding microvessels (d) were observed. (f-j) Results at 4 weeks after spinal cord injury in animals treated with angiogenic microspheres. (g) Higher magnification of the area in the rectangle in (f). Abundant Ki67-positive cells (h) and IB4-binding microvessels (i) were observed, and most Ki67-positive cells surrounded microvessels. (k-o) Results at 8 weeks after spinal cord injury in animals treated with angiogenic microspheres. (l) Higher magnification of the area in the rectangle in (k). Ki67-positive cells (m) were still observed near blood vessels (n). Scale bar = 250 μm in (a), (f), and (k); or 75 μm in (b), (c), (d), (e), (g), (h), (i), (j), (l), (m), (n), and (o).

**Supplementary video legend:**

**Supplementary videos S1-S2: Tractography videos of the spinal cord of rats** **at 12 weeks after spinal cord injury.** Tractography using diffusion tensor imaging (DTI) data allowed 3D modeling of neural tracts. These visualizations were prepared from 360, 1-degree slices by TrackVis to allow visualization from all possible angles. Tractography of animals treated with empty microspheres is shown in Supplementary Video S1, corresponding to the picture in Fig. 7a. Tractography of animals treated with angiogenic microspheres is shown in Supplementary Video S2, corresponding to the picture in Fig. 7d. Both videos show defects in the dorsal spinal cord at 12 weeks after spinal cord injury. They also show smaller lesion volume and more numerous continuous fibers in animals treated with angiogenic microspheres.
